# Heat activates the AAA+ HslUV protease by melting an axial autoinhibitory plug

**DOI:** 10.1016/j.celrep.2020.108639

**Published:** 2021-01-19

**Authors:** Vladimir Baytshtok, Xue Fei, Tsai-Ting Shih, Robert A. Grant, Justin C. Santos, Tania A. Baker, Robert T. Sauer

**Affiliations:** 1 Department of Biology, Massachusetts Institute of Technology, Cambridge, MA 02139, USA; 2 These authors contributed equally; 3 Senior author; 4 Present address: Structural Biology Program, Sloan Kettering Institute, Memorial Sloan Kettering Cancer Center, New York, NY 10065, USA; 5 MD/PhD Program, Emory University School of Medicine, 100 Woodruff Circle, Atlanta, GA 30322, USA; 6 Lead Contact

## Abstract

At low temperatures, protein degradation by the AAA+ HslUV protease is very slow. New crystal structures reveal that residues in the intermediate domain of the HslU_6_ unfoldase can plug its axial channel, blocking productive substrate binding and subsequent unfolding, translocation, and degradation by the HslV_12_ peptidase. Biochemical experiments with wild-type and mutant enzymes support a model in which heat-induced melting of this autoinhibitory plug activates HslUV proteolysis.

## In Brief

Baytshtok et al. demonstrate that the activity of HslUV, a AAA+ heat shock protease, is regulated by thermal melting of its autoinhibitory axial plug, which activates ATP hydrolysis, substrate binding, and energy-dependent proteolysis and ensures that robust protein degradation by HslUV occurs only at elevated temperatures in the cell.

## Graphical Abstract

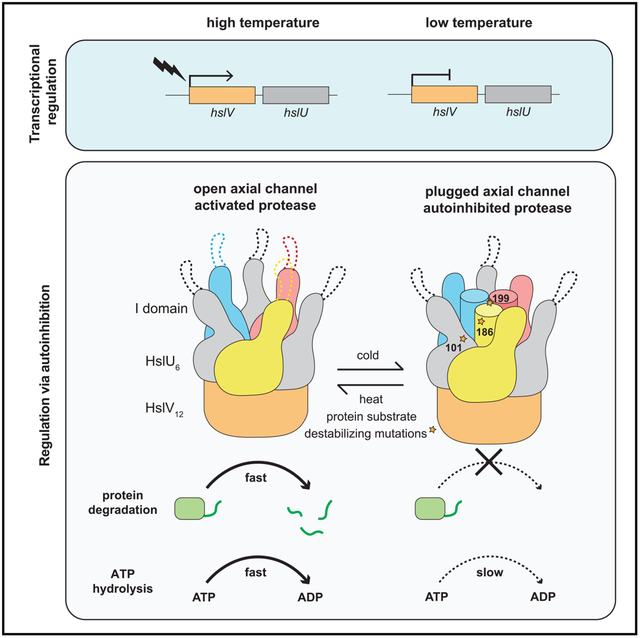

## INTRODUCTION

**Figure 1.**
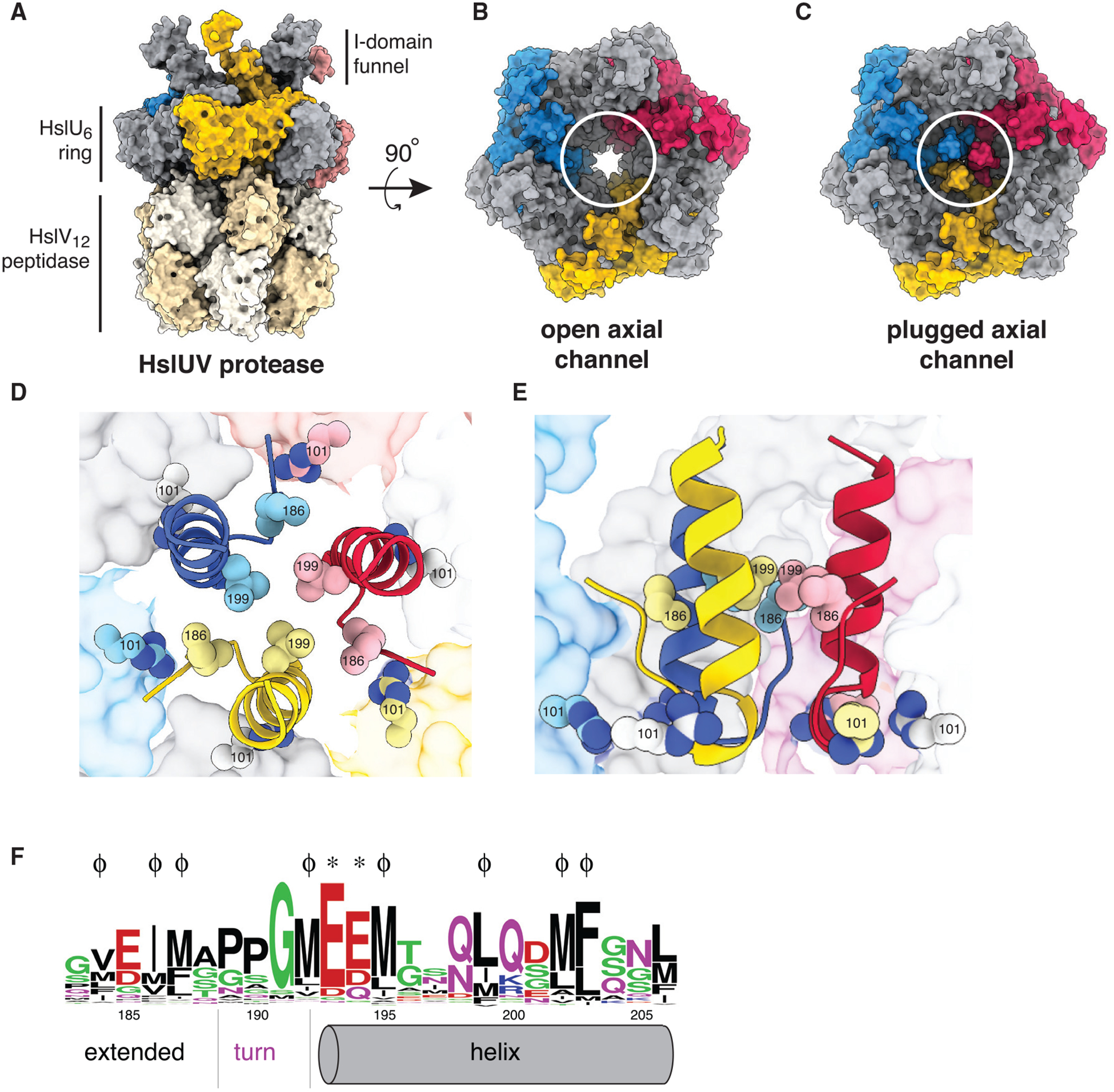
HslUV with open and closed axial channels (A) Surface-representation side view of the HslUV protease (PDB: 5JI3). (B) Surface-representation top view of open-channel HslUV structure (PDB: 5JI3). (C) Surface-representation top view of plugged-channel HslUV structure (PDB: 6PXI). (D) Top view of axial plug (PDB: 6PXI) in cartoon representation. The side chains of Arg^101^, Ile^186^, and Leu^199^ are shown as spheres. The axial channel of HslU is shown in surface representation. (E) Cutaway side view of axial plug (PDB: 6PXI) in cartoon representation with Arg^101^, Ile^186^, and Leu^199^ shown as spheres. (F) WebLogo representation of conservation of plug residues. ϕ indicates positions of hydrophobic residues (Val^184^, Ile^186^, Met^187^, Met^192^, Met^195^, Leu^199^, Met^202^, and Phe^203^) that engage in packing within the plug; asterisk (*) indicates the positions of negatively charged residues (Glu^193^ and Glu^194^) that appear to form favorable electrostatic interactions with Arg^101^ and Lys^293^ in the axial channel of the HslU hexamer.

By degrading incomplete, unfolded, or unneeded proteins, intracellular AAA+ proteases serve important roles in protein-quality control, often removing proteins damaged by heat or other destabilizing stresses (Sauer and Baker, 2011). HslUV consists of one or two AAA+ HslU_6_ ring hexamers and the double-ring HslV_12_ peptidase (Figure 1A; Rohrwild et al., 1997; Sousa et al., 2000; Wang et al., 2001). In *Escherichia coli*, expression of the HslU and HslV enzymes increases ~10-fold at high temperatures (Rohrwild et al., 1996), and HslUV proteolysis is substantially faster at high than low temperatures *in vitro* (Burton et al., 2005). ATP-dependent proteolysis requires HslU_6_ to bind the degrons of target proteins in its axial channel and then unfold and translocate the substrate into the proteolytic chamber of HslV_12_ (Sundar et al., 2012). To reach the axial channel of the HslU hexamer, substrates must transit a funnel-like structure formed by segments of its intermediate (I) domain (Figures 1A and 1B; Sousa et al., 2000; Wang et al., 2001). Both the I-domain and this funnel-like structure are unique to the HslU subfamily of AAA+ unfoldases (Sauer and Baker, 2011). An I-domain L199Q mutation enhances degradation of some protein substrates and the rate of ATP hydrolysis (Baytshtok et al., 2016), possibly by relief of autoinhibition. However, the molecular mechanism by which Leu^199^ and surrounding residues affect proteolysis and ATP hydrolysis is unknown, as amino acids 177–212 are disordered in known structures (Bochtler et al., 2000; Sousa et al., 2000; Wang et al., 2001). Here, we present evidence for a model in which HslU hexamers are autoinhibited at low temperatures by a trimeric I-domain plug that blocks the axial channel but melts at high temperatures to activate proteolysis.

## RESULTS AND DISCUSSION

### Autoinhibited structures of HslU and HslUV

We determined structures for three new crystal forms of *E. coli* HslU or HslUV by molecular replacement (Table 1).

Our new structures were similar to previous ones with the notable exception that a trimeric plug, formed by residues 183–206 of the I-domain, completely blocked the axial channel of the HslU ring (Figures 1C–1E). Because protein substrates must access the axial channel of HslU to be engaged, unfolded, and translocated into the peptidase chamber of HslV, this plug would inhibit substrate degradation. The plug consisted of amino acids from three of the six HslU subunits (Figures 1C–1E), each comprising an extended region (residues 183–188), a sharp turn (residues 189–191), and an *α* helix (residues 192–206). We could not reliably model connections between plug elements and other regions of the I-domain in specific subunits. Importantly, however, a crystallographic 3-fold axis related plug elements in the 6PXI structure, indicating that I-domain elements from three alternating subunits of the HslU hexamer must form the plug.

The three *α* helices that comprise the plug center packed together via hydrophobic contacts involving the side chains of Met^192^, Met^195^, Leu^199^, and Phe^203^. Hence, replacing the nonpolar Leu^199^ with a polar Gln^199^ in the ^L199Q^HslU variant should destabilize helix-helix packing and plug stability, thereby reducing autoinhibition and providing a structural rationale for the ability of ^L199Q^HslUV to degrade some protein substrates more rapidly than wild-type HslUV (^WT^HslUV; Baytshtok et al., 2016). In prior biochemical experiments, the subtilisin and chymotrypsin endoproteases were found to cleave sites within the plug region of ^L199Q^HslU faster than in ^WT^HslU (Baytshtok et al., 2016), supporting a model in which destabilization of the mutant plug results in partial melting and increased susceptibility to proteolytic cleavage. Additional hydrophobic packing contacts within the plug included Val^184^ and Ile^186^ from the extended region, which occupy peripheral grooves between plug helices, and Met^202^ from the helix. Notably, residues that form hydrophobic interactions within the plug are highly conserved in HslU orthologs (Figure 1F).

A Pro^189^-Pro^190^-Gly^191^ tripeptide formed a sharp turn between the extended region and *α* helix of the plug. The negatively charged side chains of Glu^193^ and Glu^194^ following this turn were also close to the positively charged side chains of Arg^101^ (94% conserved) and Lys^293^ (92% conserved) in the HslU axial channel, suggesting that favorable electrostatic interactions also stabilize the plugged conformation. Conservation of the plug residues that form the turn and electrostatic interactions within the HslU channel (Figure 1F) provide additional support for a model in which the autoinhibited conformation is functionally important.

### Electron microscopy of closed- and open-channel HslU structures

**Figure 2.**
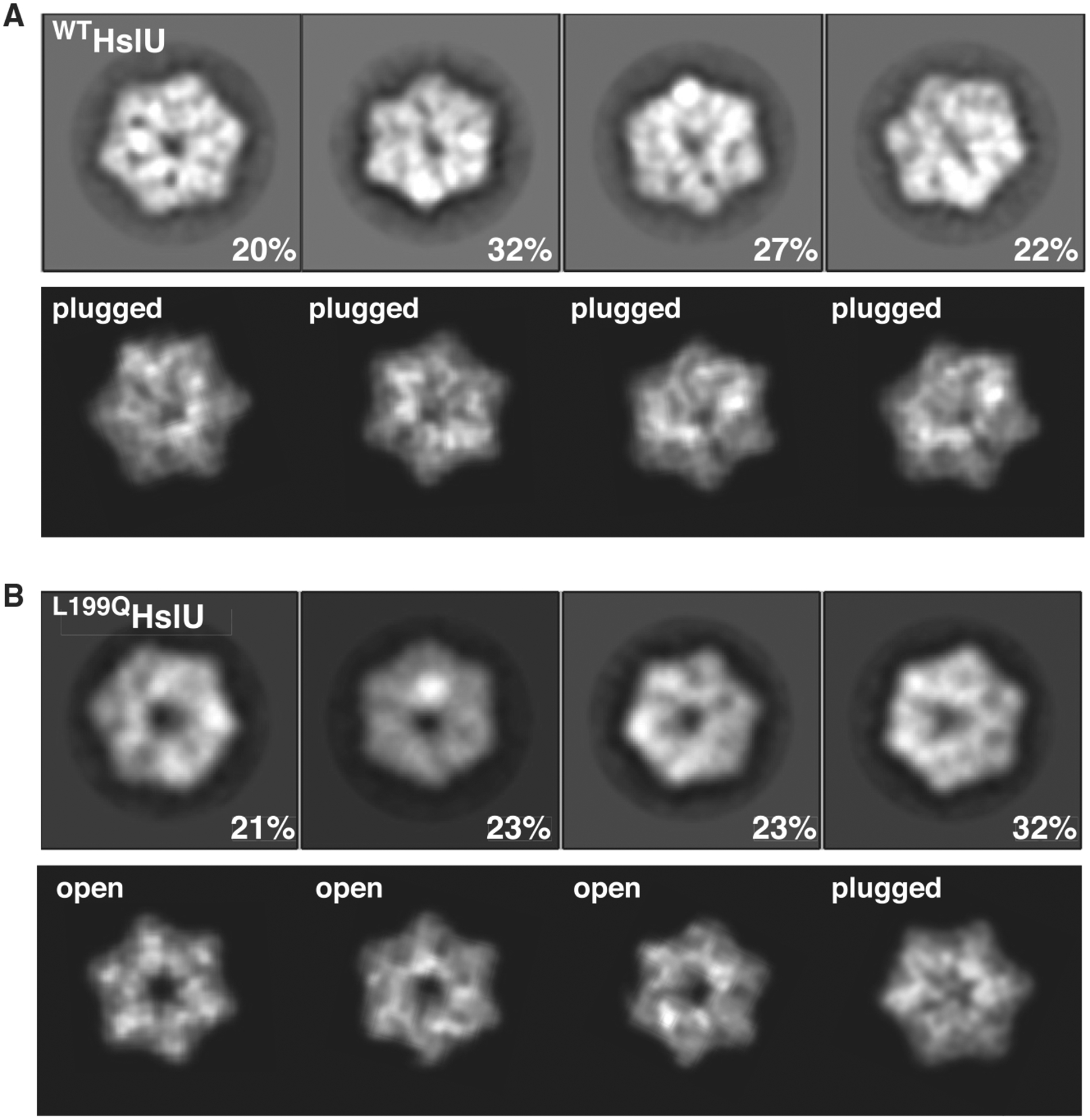
Effects of L199Q mutation on HslU channel size (A) Class-average negative-stain electron microscopy (EM) images of ^WT^HslU (top row) compared with projections of a plugged hexamer (PDB: 6PXK) filtered to 15-Å resolution (bottom row). Samples were prepared at room temperature. (B) Class-average images of ^L199Q^HslU (top row) compared with filtered images of an open-channel hexamer (PDB: 5JI3) or the plugged hexamer (PDB: 6PXK).

To investigate channel accessibility in ^WT^HslU and ^L199Q^HslU in a non-crystalline environment, we used negative-stain electron microscopy. Both enzyme samples were equilibrated at room temperature prior to application to grids and staining. Class-average images of ^WT^HslU, viewed approximately down the axis of the AAA+ ring, showed a shallow axial depression or blocked channel (Figure 2A, top row), similar to projections of plugged crystallographic HslU hexamers filtered to 15 Å (Figure 2A, bottom row). Most class averages for ^L199Q^HslU hexamers (Figure 2B, top row), by contrast, resembled open-channel crystal structures filtered to 15 Å (Figure 2B, bottom row). These results provide further evidence that the axial channel of ^WT^HslU at room temperature is plugged in most enzymes and that the L199Q mutation shifts the equilibrium to favor the open-channel conformation.

### Plug stability affects temperature-dependent activity

**Figure 3.**
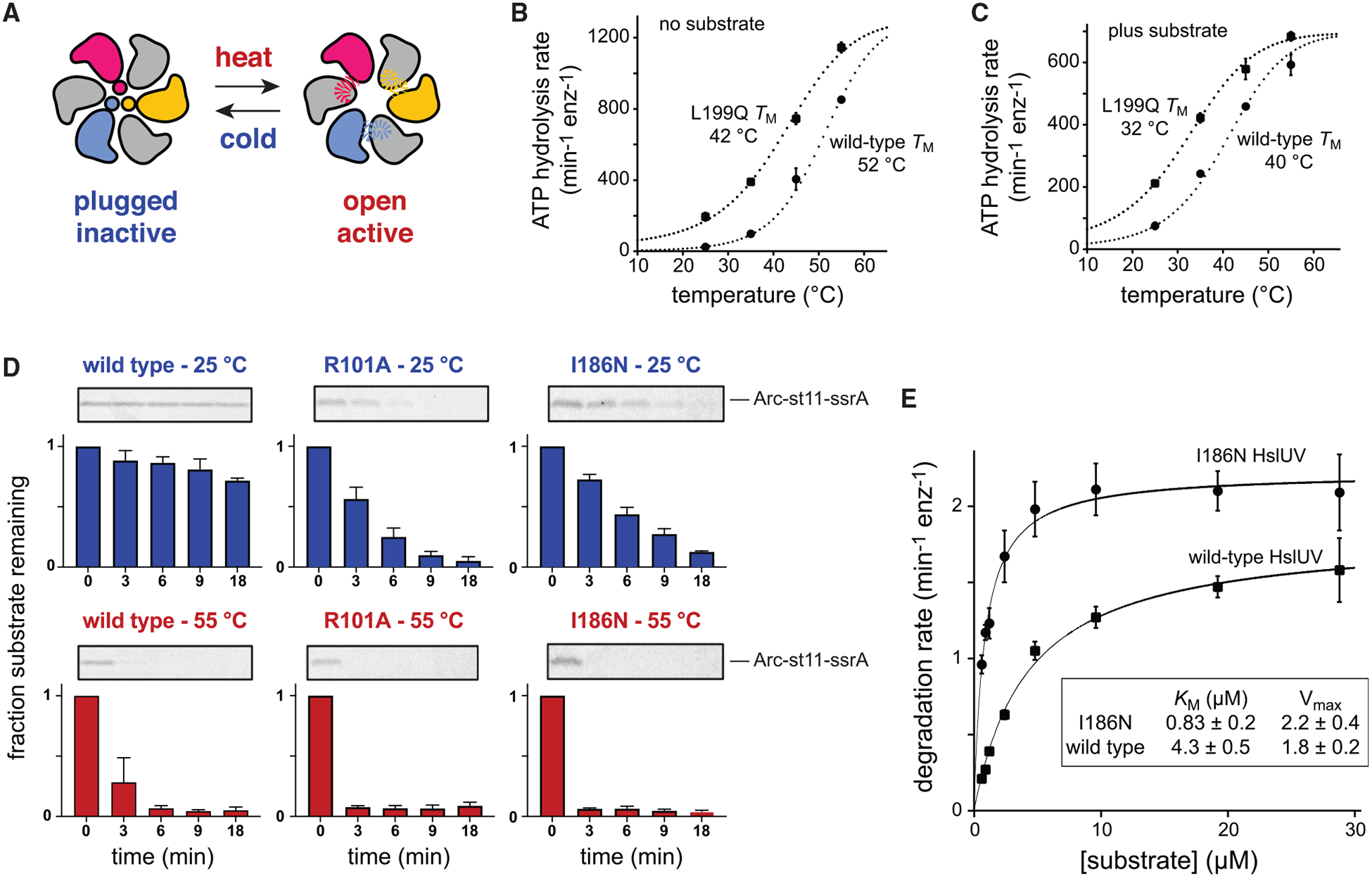
Activities of ^WT^HslUV and mutants with destabilized plugs (A) Model for activation of HslU by thermal melting of the autoinhibitory plug. (B) Temperature dependence of ATP hydrolysis by ^WT^HslUV or ^L199Q^HslUV (0.3 μM HslU_6_, 0.9 μM HslV_12_). Values are averages (±SD) of three independent experiments. The dotted lines are fits to melting curves. (C) Same as in (B) but in the presence of the ^I37A^Arc-^cp6^GFP-st11-ssrA substrate (50 μM). (D) SDS-PAGE was used to assay the kinetics of degradation of the Arc-st11-ssrA substrate (5 μM) at 25°C or 55°C by ^WT^HslUV, ^R101A^HslUV, or ^I186N^HslUV (0.3 μM HslU_6_, 0.9 μM HslV_12_). In each subpanel, a representative gel is shown at the top, and the mean (±SD) of substrate remaining in three independent replicates is shown in the graph below. (E) Michaelis-Menten plots and steady-state kinetic parameters for ^I37A^Arc-^cp6^GFP-st11-ssrA degradation at 37°C by ^WT^HslUV or ^I186N^HslUV (0.3 μM HslU_6_, 0.9 μM HslV_12_). Values plotted are averages (±SD) of three independent replicates.

We propose that high temperature destabilizes the plug and favors the active open-channel conformation of HslUV, whereas low temperature stabilizes the inactive plugged-channel conformation (Figure 3A). As a first test of this model, we measured the rates of ATP hydrolysis by ^WT^HslUV and ^L199Q^HslUV at 25°C, 35°C, 45°C, and 55°C without or with the ^I37A^Arc-^cp6^GFP-st11-ssrA protein substrate (Figures 3B and 3C). These data fit well to melting curves calculated assuming that the high-temperature conformations of ^WT^HslUV and ^L199Q^HslUV had the same ATPase activity, whereas the low-temperature conformations were inactive in ATP hydrolysis. Without protein substrate, the fitted temperatures of half-maximal activity (*T*_M_) were 42°C for ^L199Q^HslUV and 52°C for ^WT^HslUV (Figure 3B). Protein substrate reduced the *T*_M_ values by 10°C–12°C for both enzymes (Figure 3C), indicating that binding of the ^I37A^Arc-^cp6^GFP-st11-ssrA substrate preferentially stabilizes the active enzyme conformation. The lower *T*_M_ values for ^L199Q^HslUV than for ^WT^HslUV, both with and without protein substrate, strongly support our model that plug melting results in temperature-dependent relief of autoinhibition.

As a second test of our temperature-dependent activation model, we used SDS-PAGE to measure HslUV degradation of an Arc-st11-ssrA substrate at 25°C or 55°C (Figure 3D). As expected from findings of a previous study (Burton et al., 2005), degradation by the WT enzyme was very slow at 25°C and much faster at 55°C. We also assayed degradation of the same substrate by ^R101A^HslUV, constructed to remove favorable electrostatic interactions between Arg^101^ in the axial channel and glutamates in the autoinhibitory plug, and by ^I186N^HslUV, which replaces a hydrophobic residue that normally stabilizes plug packing with a polar residue (Figures 1D and 1E). At 25°C, both the ^R101A^HslUV and ^I186N^HslUV enzymes degraded Arc-st11-ssrA substantially faster than did ^WT^HslUV (Figure 3D), as expected by our model. At 55°C, all three enzymes degraded Arc-st11-ssrA more rapidly than at the lower temperature, with ^WT^HslUV degradation being slower than degradation by the mutant enzymes. In each case, faster substrate degradation at the higher temperature probably results from a combination of reduced autoinhibition and reduced stability of the native portion of the Arc-st11-ssrA substrate. Importantly, these results support a model in which the R101A and I186N mutations destabilize the autoinhibited conformation at low temperatures and thereby increase protease activity relative to the WT enzyme.

As a third test of our model, we determined steady-state degradation rates of different concentrations of the ^I37A^Arc-^cp6^GFP-st11-ssrA substrate at a temperature (37°C) at which both ^WT^HslUV and ^I186N^HslUV have substantial activity and fit the data to the Michaelis-Menten equation to determine *K*_M_ and V_max_ values (Figure 3E). At low substrate concentrations, degradation by ^I186N^HslUV was ~6-fold faster than by ^WT^HslUV, largely as a consequence of a tighter *K*_M_ for the mutant (Figure 3E). By contrast, degradation of high concentrations of this substrate by ^I186N^HslUV was only ~1.2-fold faster than by ^WT^HslUV. These results support a model in which plug destabilization, as a consequence of reduced hydrophobic packing, makes the axial channel accessible in a larger fraction of the population of I186N mutant enzymes at low substrate concentrations, thereby resulting in tighter substrate binding and faster degradation. Very high substrate concentrations, in turn, appear to shift the equilibrium toward the open-channel conformation of ^WT^HslUV, thereby reducing the activity difference between the mutant and WT enzymes.

### Conclusions and biological inferences

We have identified a new conformation of the HslUV protease in which part of the I-domain forms a plug that blocks the axial channel of the AAA+ HslU ring and prevents productive substrate engagement and thus degradation. This plugged-channel inactive conformation is in dynamic equilibrium with an open-channel active conformation, with higher temperature and/or higher protein-substrate concentrations favoring the active conformation.

In cells, direct thermal activation of HslUV by melting of the autoinhibitory plug at heat shock temperatures (Figure 3A) would increase protease activity almost immediately. Temperature-induced expression increases of HslU and HslV would then further amplify HslUV proteolytic capacity. Following a return to lower temperatures, levels of the HslUV enzyme would be expected to remain high for several generations before reduced expression and cell division allowed a return to the low-temperature steady state. During this transition from high to low temperatures, increased plug-mediated autoinhibition could contribute to cell fitness by minimizing rogue HslUV proteolysis and/or reducing excessive ATP hydrolysis.

## STAR★METHODS

### RESOURCE AVAILABILITY

#### Lead Contact

Requests for information and resources should be directed to and will be fulfilled by the Lead Contact, Robert T. Sauer (bobsauer@mit.edu).

#### Materials Availability

This study generated new His_6_-tagged mutants of *E. coli* HslU containing the R101A and I186N mutations. Plasmids expressing these mutant enzymes are available from the lead contact upon request without restriction.

#### Data and Code Availability

Coordinates, structure factors and electron-density maps for the 6PXI, 6PXL, and 6PXK crystal structures are available from the RCSB Protein Data Bank (https://www.rcsb.org/). The biochemical data and electron micrographs supporting the current study are available from the Lead Contact upon request.

### EXPERIMENTAL MODEL AND SUBJECT DETAILS

Proteins were expressed in *E. coli* strain X90 (λDE3) *sly*D::*kan hsl*UV::*tet*). No additional biological strains were used in this work.

### METHOD DETAILS

Genes encoding H_6_-tagged variants of *E. coli* HslU, ^E257Q^HslU, ^L199Q^HslU, ^I186N^HslU, ^R101A^HslU, HslV, ^I37A^Arc-^cp6^GFP-st11-ssrA, and Arc-st11-ssrA were expressed from pET12b or pET21b vectors (Novagen) in *E. coli* strain X90 (λDE3) *sly*D::*kan hsl*UV::*tet* and proteins were purified as described (Baytshtok et al., 2016).

Crystals of HslU were grown at 4°C by the hanging-drop method after mixing 0.5 μL of selenomethionine-labeled H_6_-tagged HslU (15 mg/mL) in 15 mM Tris (pH 7.5), 100 mM NaCl, 20 mM MgCl_2_, 0.2 mM EDTA, and 5 mM ATPγS with an equal volume of well solution containing 100 mM Tris (pH 7.5), 18% PEG-3350, and either 0.1 M (PDB code 6PXK) or 0.2 M (PDB code 6PXL) ammonium sulfate. For cryo-protection, PEG-3350 was increased to 20% and 10% MPD was added to the base-well solution. HslUV crystals were grown in the same way except the protein solution contained ^E257Q^HslU (10 mg/mL) plus HslV (9.6 mg/mL) and the well solution contained 0.1 M Bis-Tris (initial pH 5.5), 1.85 M ammonium sulfate, 5% glycerol, and 5 mM ATPγS. For cryo-protection, glycerol was increased to 25%. Diffraction data for the HslUV crystals were collected on our home source (Rigaku MicroMax-007HF with a Saturn 944 detector) and for the HslU crystals at the Advanced Photon Source (APS) beamline 24-ID-E and processed using HKL2000 (Otwinowski and Minor, 1997) or XDS (Kabsch, 2010). Structures were solved by molecular replacement with PHASER (McCoy et al., 2007) using search models consisting of HslU hexamers alone or HslUV (PDB code 5JI3; Baytshtok et al., 2016). Anomalous difference maps – showing the selenomethionine side chains of residues 187, 192, 195, and 202 – were used to assist model building into plug density. Phenix was used to refine structures (Adams et al., 2010), Coot was used for model building (Emsley et al., 2010), and MolProbity was used to assess model geometry (Chen et al., 2010).

Negative-stain EM experiments were performed as described (Baytshtok et al., 2016), using WT ^WT^HslU or ^L199Q^HslU (1.5 μM) and ATPγS (5 mM) in 20 mM HEPES (pH 7.5), 5 mM MgCl_2_, 500 mM NaCl, 10% glycerol (v/v), and 0.032% Igepal CA-630. Particles with tilted six-fold symmetry axis were removed by two rounds of 2D classification. For ^WT^HslU, 10927 particles from 23 micrographs were used to generate representative 2D class averages; for ^L199Q^HslU, 5567 particles from 20 micrographs were used. The e2pdb2mrc and e2project3d utilities in EMAN2 (Tang et al., 2007) were used to generate 2D projections from PDB files and Relion 3.0.8 (Scheres, 2012) was used for 2D classification.

Degradation and ATPase assays were performed in 25 mM HEPES (pH 7.5), 5 mM KCl, 20 mM MgCl_2_, 10% glycerol, and 0.032% Igepal CA-630 as described (Baytshtok et al., 2016). ATPase rates at different temperatures were measured using a Spectramax M5 plate reader (Molecular Devices) by using an NADH-coupled assay (Nørby, 1988) with or without 50 μM ^I37A^Arc-^cp6^GFP-st11-ssrA. Using the Solver tool of Microsoft Excel, the temperature dependencies of ATPase rates were globally fitted to the equation max/(1+exp(ΔH/R·(1/*T*_M_-1/*T*))), where max is the maximum ATPase rate, *T* is the temperature in Kelvin, *T*_M_ is the temperature at 50% activity, ΔH is the enthalpy at *T*_M_, and R is the universal gas constant. Degradation of ^I37A^Arc-^cp6^GFP-st11-ssrA by ^WT^HslUV or ^I186N^HslUV was monitored by loss of fluorescence (excitation 467 nm; emission 511 nm). For degradation of Arc-st11-ssrA (5 μM) by wild-type or mutant enzymes (0.3 μM HslU_6_, 0.9 μM HslV_12_), proteins were preincubated for 1 min at 25°C or 55°C before adding an ATP regeneration system (2.5 mM ATP, 7.5 mM phosphoenolpyruvate, 1 mM NADH, 18.8 U/ml pyruvate kinase, 21.5 U/ml lactate dehydrogenase) to initiate degradation. Aliquots were removed at different times, quenched by addition of Tricine sample buffer (Bio-Rad) with β-mercaptoethanol, and flash frozen in liquid nitrogen. Samples were then boiled for 5 min and electrophoresed on 15% Tris-Tricine SDS-PAGE gels. After electrophoresis, gels were stained for 10 min using a Coomassie-blue solution, destained overnight in water, scanned using a Typhoon FLA 9500 Imager (GE Healthcare), and the amount of substrate remaining at each time point was quantified using ImageQuant TL (GE Healthcare).

### QUANTIFICATION AND STATISTICAL ANALYSIS

Crystallographic unit-cell parameters, data completeness and redundancy, CC_1/2_ values, and merging R-factors (Table 1) were obtained using HKL2000 (Otwinowski and Minor, 1997), which was used to index, integrate, and scale the diffraction data. Model-refinement statistics (*R*_work_, *R*_free_; Table 1) were obtained from the phenix.refine program in Phenix (Adams et al., 2010) and are shown in Table 1. Statistics for the quality of the final crystallographic models (Table 1) were determined using MolProbity (Chen et al., 2010). Biochemical experiments were performed using a minimum of three independent replicates. Biochemical data were plotted as averages ± one standard deviation (SD), as described in the Figure 3 legend, using Prism (GraphPad). The errors for *K*_M_ and V_max_ (Figure 3E) were calculated by non-linear least-squares fitting to the Michealis-Menten equation using Prism (GraphPad).

## Figures and Tables

**Table 1.** Crystallographic statistics

PDB entry	6PXI	6PXL	6PXK
Protein	HslUV	HslU	HslU
Resolution (Å)	3.45	3.74	3.65
Space group	P32_1_	C2	P2
Unit cell			
A (Å)	168.60	414.59	200.03
B(Å)	168.60	92.33	91.20
C(Å)	162.89	200.85	201.80
α	90°	90°	90°
β	90°	99.43°	99.43°
γ	120°	90°	90°
No. of unique reflections	29,212	68,314	15,427
Redundancy	2.2	3.8	5.0
Completeness (%)	81.7 (59.3)	95.3 (77.1)	98.4 (92.5)
CC-1/2	0.985 (0.593)	0.981 (0.669)	0.998 (0.609)
R_sym_	0.114	0.121	0.105
R_pim_	0.081	0.068	not calculated
R_work_^/^R_free_	0.257/0.285	0.220/0.265	0.223/0.271
MolProbity score (percentile)	1.04 (100)	1.31 (100)	1.30 (100)
Clash score (percentile)	2.54 (100)	5.71 (100)	5.9 (100)
Ramachandran outliers (%)	0	0	0
Ramachandran favored (%)	98.58	98.15	99.26
Bad bonds/angles	0/0	0/0	0/0
Cβ deviations	0	0	0

Completeness and CC-1/2 values in parentheses are for the highest resolution shell.

**Table:** KEY RESOURCES TABLE

REAGENT or RESOURCE	SOURCE	IDENTIFIER
Bacterial Strains		
X90 (λDE3) *slyD::kan hslUV::tet*	Baytshtok et al., 2016	N/A
Proteins and Enzymes		
HslU (His_6_ tagged)	Bochtler et al., 2000	N/A
^L199Q^HslU (His_6_ tagged)	Baytshtok et al., 2016	N/A
^R101A^HslU (His_6_ tagged)	This study	N/A
^i186N^Hsiu (His_6_ tagged)	This study	N/A
^e257Q^Hsiu (His_6_ tagged)	Yakamavich et al., 2008	N/A
HslV (His_6_ tagged)	Bochtler et al., 2000	N/A
^I37A^Arc-^cp6^GFP-st11-ssrA	Baytshtok et al., 2016	N/A
Arc-st11-ssrA	Milla et al., 1993	N/A
pyruvate kinase	Sigma-Aldrich	N/A
lactate dehydrogenase	Sigma-Aldrich	N/A
Deposited Data		
^E257Q^HsU-HslV-ADP, plugged	This study	PDB: 6PXI
^WT^HslU- ADP, plugged	This study	PDB: 6PXL
^WT^HslU- ADP, plugged	This study	PDB: 6PXK
Software and Algorithms		
Coot	Emsley et al., 2010	N/A
ChimeraX	Goddard et al., 2018	N/A
Ctffind3	Mindell and Grigorieff, 2003	N/A
EMAN2	Tang et al., 2007	N/A
HKL2000	Otwinowski and Minor, 1997	N/A
Phenix	Adams et al., 2010	N/A
PyMOL	Schrodinger, LLC	N/A
Relion	Scheres, 2012	N/A
XDS	Kabsch, 2010	N/A

## Highlights

- A trimeric structure can plug the axial channel of the AAA+ HslU ring hexamer
- This autoinhibitory plug is observed in crystal structures and by electron microscopy
- Temperature-induced melting of the plug activates ATP hydrolysis and proteolysis
- Low-temperature autoinhibition limits rogue degradation after recovery from heat shock
